# Structural and Chemical Degradation of Archeological Wood: Synchrotron XRD and FTIR Analysis of a 26th Dynasty Egyptian Polychrome Wood Statuette

**DOI:** 10.3390/polym18020258

**Published:** 2026-01-17

**Authors:** Dina M. Atwa, Rageh K. Hussein, Ihab F. Mohamed, Shimaa Ibrahim, Emam Abdullah, G. Omar, Moez A. Ibrahim, Ahmed Refaat

**Affiliations:** 1 Department of Laser Interaction with Matters, Laser Institute for Research and Applications, Beni-Suef University, Beni-Suef P.O. Box 62517, Egypt; ghada.omar@lira.bsu.edu.eg; 2 Physics Department, College of Science, Imam Mohammad Ibn Saud Islamic University (IMSIU), Riyadh 11623, Saudi Arabia; rahussein@imamu.edu.sa (R.K.H.); maimohammed@imamu.edu.sa (M.A.I.); 3 Department of Anthropology, Faculty of Postgraduate African Studies, Cairo University, Giza P.O. Box 12613, Egypt; ihab_fathi79@faps.cu.edu.eg; 4 Department of Botany, Faculty of Science, Cairo University, Giza P.O. Box 12613, Egypt; shimaa_sa3d53@yahoo.com; 5 Pyramids Area, Projects Sector, Ministry of Tourism and Antiquities, Giza P.O. Box 12556, Egypt; emamabdallah@gmail.com; 6 Spectroscopy Department, National Research Centre, 33 El-Bohouth St., Dokki, Giza P.O. Box 12622, Egypt; am.refaat@nrc.sci.eg; 7 Molecular Modeling and Spectroscopy Laboratory, Centre of Excellence for Advanced Science, National Research Centre, 33 El-Bohouth St., Dokki, Giza P.O. Box 12622, Egypt

**Keywords:** synchrotron X-ray diffraction, wood degradation, 26th Dynasty, polychrome wooden sculpture, ancient Egyptian pigments, Egyptian blue, atacamite, archaeometry, crystalline index of cellulose, natural polymers

## Abstract

This study investigates a 26th Dynasty Ptah–Sokar–Osiris wooden statuette excavated from the Tari cemetery, Giza Pyramids area, to decode ancient Egyptian manufacturing techniques and establish evidence-based conservation strategies of such wooden objects. Using minimal sampling (1.0–2.0 mm^2^), integrated XRF, synchrotron-based X-ray diffraction, FTIR, and confocal microscopy distinguished original technological choices from burial-induced alterations. The 85 cm *Vachellia nilotica* sculpture exhibits moderate structural preservation (cellulose crystallinity index 62.9%) with partial chemical deterioration (carbonyl index 2.22). Complete pigment characterization identified carbon black, Egyptian Blue (cuprorivaite, 55 ± 5 wt %), atacamite-dominated green (65 ± 5 wt %) with residual malachite (10 ± 2 wt %), orpiment (60 ± 5 wt %), red ochre (hematite, 60% ± 5 wt %), white pigments (93 ± 5 wt % calcite), and metallic gold (40 ± 5 wt %). Confocal microscopy revealed sophisticated multi-pigment mixing strategies, with black carbon systematically blended with chromophores for nuanced color effects. Atacamite predominance over malachite provides evidence for chloride-mediated diagenetic transformation over 2600 years of burial. Consistent calcite detection (~ 20–45%) across colored layers confirms systematic ground layer application, establishing technological baseline data for 26th Dynasty Lower Egyptian workshops. Near-complete organic binder loss, severe lignin oxidation, and ongoing salt-mediated mineral transformations indicate urgent conservation needs requiring specialized consolidants, paint layer stabilization, and controlled environmental storage. This investigation demonstrates synchrotron methods’ advantages while establishing a minimally invasive framework for studying polychrome wooden artifacts.

## 1. Introduction

Ancient Egyptian polychrome wooden artifacts are invaluable sources of knowledge about ancient civilizations [1]. The study of such objects serves key aims: identifying original materials and manufacturing techniques, understanding degradation processes, establishing authentication criteria for undocumented artifacts, and linking material evidence with archeological and historical contexts [1,2]. Among the diverse categories of Egyptian painted wooden artifacts, funerary statuettes of the composite deity Ptah–Sokar–Osiris represent a particularly significant class of objects that emerged during the late New Kingdom (ca. 1070 BCE) and became standard components of elite burial assemblages throughout the Late Period (712–332 BCE) and Ptolemaic era (332–30 BCE) [3,4,5]. Despite their abundance and religious significance, the technological features of these statuettes—especially wood-working methods, pigment compositions, preparation layers, and regional workshop practices—remain only partially documented in archaeometric studies [1,2,3].

In addition to their artistic and ritual importance, these objects must be examined not only at the level of the painted layers but also through the perspective of wood science, as wood is a complex natural polymeric composite composed mainly of cellulose, hemicellulose, and lignin [6,7,8]. As an organic, hygroscopic material, wood is highly susceptible to physicochemical transformations during both burial and long-term aging [9]. Over centuries in tomb environments, fluctuating humidity, microbial activity, and interactions with burial materials can cause gradual cellulose depolymerization, selective hemicellulose loss, reduced microfibrillar order, and structural alterations in lignin [9,10]. These processes reduce cellulose crystallinity in most cases, alter mechanical strength, and can ultimately reshape the microstructural integrity of the wooden core [9]. Such deterioration pathways influence not only the stability of the carving itself but also the performance of applied artistic layers (ground preparations, pigments, and binding media), which depend on the wood’s dimensional and chemical stability [6,7,8,9,10,11]. Understanding the hierarchical degradation of wood as a natural polymer is therefore essential for assessing the condition of such polychrome objects and designing conservation strategies suited to their composite nature. Achieving this requires complementary analytical approaches that integrate elemental, molecular, and crystallographic methods to evaluate deterioration in the paint layers, track changes in cellulose, hemicellulose, and lignin, and determine their effects on both the wooden substrate and the overlying artistic layers [7,8,9,10,11,12,13,14].

Such integrated analytical techniques may contain XRF to provide elemental composition of painted colors, XRD to reveal pigment mineralogy and quantify wood degradation through cellulose crystallinity index (CrI) measurements, and FTIR to deliver molecular-level identification of organic and inorganic components. Collectively with optical microscopy, they enable the assessment of both original artistic materials and deterioration mechanisms [6,7,8,9,10,11,12,13,15,16,17,18].

Archaeometric research on ancient Egyptian polychrome wooden artifacts has advanced considerably through the application of the abovementioned non-destructive and minimally invasive techniques (XRF, XRD, FTIR, and optical microscopy), enabling improved identification of pigments, preparation layers, and wood species, as well as insight into manufacturing technologies and degradation processes [1,2,6]. Parallel studies in wood science have clarified key deterioration mechanisms, including cellulose depolymerization, crystallinity loss, lignin oxidation, and changes in hygroscopic behavior driven by burial and aging conditions [7,8,9,10,11,12,13,16,18,19,20].

In recent studies, FTIR and XRD analyses of aged cedar wood have revealed progressive crystallinity decline from modern to historic samples, reflecting increased cellulose amorphization [7,12,19]. This reduced CrI correlates with microstructural collapse, pore enlargement, and compromised structural integrity, potentially affecting wood–paint interface stability and the adhesion of ground layers, pigments, and binders in polychrome sculptures [11,19,20].

Nevertheless, many investigations rely on single or partially integrated methods, limiting the ability to resolve heterogeneous, multiphase pigment systems or to correlate pigment composition with the structural and chemical state of the wooden substrate. Elemental techniques alone cannot distinguish mineral phases or assess polymer degradation, while conventional laboratory methods often require sample quantities incompatible with fragile cultural heritage objects [14,15,16,21]. Although synchrotron-based XRD offers exceptional sensitivity and spatial resolution for micro-samples, its application to Egyptian polychrome wood remains limited [22,23,24,25].

Synchrotron radiation X-ray diffraction (SR-XRD) overcomes conventional XRD’s milligram sample requirement through high photon flux, micrometer-scale resolution, and enhanced sensitivity to trace phases (<1%), enabling comprehensive analysis of microscopic archeological samples while preserving precious artifacts [19,21,22,23,24].

Our study addresses the mentioned gaps by adopting a fully integrated, multi-analytical approach that combines synchrotron-based XRD with XRF, FTIR, and confocal microscopy, enabling comprehensive characterization of pigments and wood degradation from minimal sampling [1,26,27]. This strategy enhances discrimination between original technologies and post-depositional alterations and provides robust data to inform both technological interpretation and conservation planning

In our work, we analyzed a well-provenanced 26th Dynasty Ptah–Sokar–Osiris statuette from Tari cemetery, Giza (excavated 2020), whose secure archeological context and variable preservation state provide an ideal case study for investigating wood manufacturing, pigment technology, and degradation mechanisms in Late Period polychrome wooden artifacts.

This study evaluates whether Saite artistic archaism correlates with technical continuity or innovation in workshop practices. By integrating conventional spectroscopy with synchrotron methods, we demonstrate that minimally invasive sampling can yield comprehensive material characterization, establishing reference standards for conservation protocols while advancing our understanding of ancient Egyptian craft technology and material science.

## 2. Materials and Methods

### 2.1. Archeological Context and Object Description

#### 2.1.1. Excavation Site

The Giza Plateau, located approximately 8 km southwest of Cairo, comprises the Fourth Dynasty pyramid complexes (2613–2494 BCE), the Great Sphinx, and extensive cemeteries that continued to be reused in later periods. Within this landscape, the Tari cemetery—situated southeast of the pyramids—is a limestone-constructed mass burial complex originally established during the Fourth Dynasty and subsequently reused during the Twenty-Sixth Dynasty (664–525 BCE) [28]. It was within this multi-period funerary context that the Ptah–Sokar–Osiris statuette was discovered in July 2020 during excavations directed by Dr. Mostafa Waziry. The object was found inside a double burial chamber where Late Period reuse of Old Kingdom architecture was clearly visible [28]. Tomb inscriptions record original Old Kingdom owners, including “Bahn Wai Ka” and “Nawa”, indicating elite burial status. The burial context contained sandstone and limestone fragments, loose sand deposits, and signs of episodic moisture infiltration that affected the preservation of organic materials and facilitated the formation of secondary minerals.

#### 2.1.2. Object Description

**Figure 1:**
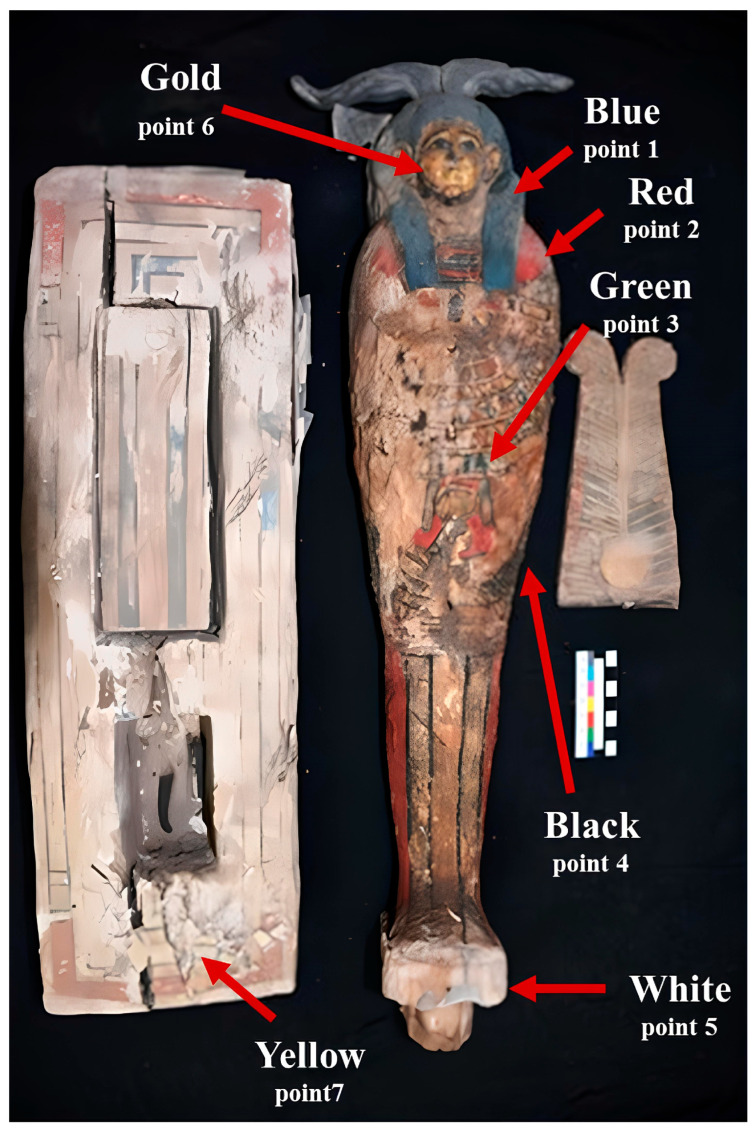
Polychrome wooden Ptah–Sokar–Osiris statuette (26th Dynasty, 85 cm) made from single-piece *Vachellia nilotica* (*Acacia nilotica*) wood with a rectangular base (77 cm × 27 cm× 10 cm). Red arrows mark sampling points where seven pigment types were identified: Egyptian Blue, hematite (red), orpiment (yellow), atacamite/malachite (green), carbon (black), calcite (white), and gold. The base cavity was empty, lacking the typical papyrus scroll. Visible degradation includes paint separation, surface friability, and salt efflorescence. Scale bar = 10 cm. Pigment sampling locations on the statuette: blue (point 1, wig), red (point 2, chest), green and black (points 3 and 4, lower body), white (point 5, remaining areas), gold (point 6, face), and yellow (point 7, base edge).

The statuette measures 85 cm in length with a shoulder width of 23 cm and is carved from a single piece of *Vachellia nilotica* (*Acacia nilotica*) wood, mounted on a rectangular base measuring 77 cm × 27 cm × 10 cm. It represents Ptah–Sokar–Osiris in anthropomorphic form, with human facial features and characteristic shroud iconography. The polychrome decoration includes blue, red, yellow, green, black, and white pigments, along with traces of gold leaf, indicative of elite patronage. Figure 1 presents a detailed image of the statuette, with arrows indicating the locations from which samples were collected.

Sampling locations are marked by red arrows, where blue pigment was sampled from point 1 on the statuette’s wig, red pigment was collected from point 2 on the statuette’s chest, and green and black pigments were collected from points 3 and 4, respectively, located on the lower part of the statuette’s body. White pigment was collected from the rest of statuette (point 5), while gold was sampled from its face (point 6) and yellow pigment was collected from point 7 on the base edge.

The paint layers exhibit varying degrees of detachment, particularly on the rectangular base, where Japanese paper has been applied to support warped areas. A rectangular cavity in the base was found empty, in contrast to typical examples of this type of deity model. Preservation conditions vary considerably: paint layers show separation, detachment, and complete loss in some regions; surfaces exhibit powdering and friability; localized biological damage is present; and efflorescent salts have accumulated in certain areas.

### 2.2. Sample Selection and Description

#### 2.2.1. Sampling Strategy

Ten color samples and one wood sample (about 1.0–2.0 mm^2^ each) were collected in accordance with cultural heritage best practices exclusively from areas that were naturally detached, deteriorated, or previously damaged [9,10]. All sampling locations were photographically documented, with spatial coordinates recorded. The sampling strategy was designed to achieve multiple objectives: comprehensive documentation of the pigment palette, characterization of the preparation layers, identification of the binding media, assessment of wood degradation, analysis of degradation products, and evaluation of the burial environment. Table 1 provides details on the description, spatial location, and material type of the analyzed samples.

#### 2.2.2. Sample Handling

Samples were individually stored in polyethylene microtubes, each labeled with a unique identifier, under controlled conditions of 25 ± 2 °C and 40 ± 5% relative humidity [29]. Wood and pigment samples were analyzed without pretreatment and stored under controlled conservation laboratory conditions prior to examination. All handling was performed under confocal microscopic observation using cleaned stainless-steel tools to minimize contamination. The analytical workflow prioritized non-destructive techniques, including XRF and confocal microscopy, prior to any invasive procedures such as SR-XRD mounting and FTIR analysis.

### 2.3. Diagnostic Methods

#### 2.3.1. X-Ray Fluorescence

X-ray fluorescence (XRF) analysis was performed in mining mode using a Thermo Scientific Niton XL3 handheld analyzer (Thermo Fisher Scientific, Waltham, MA, USA) to determine the elemental composition of each sample.

#### 2.3.2. X-Ray Diffraction Analysis

X-ray diffraction (XRD) analysis was performed at the MCX beamline of Elettra-Sincrotrone Trieste under the ICTP-Elettra Users Programme, using a monochromatic 12 keV X-ray beam (λ = 1.0332 Å) with point-focus geometry (0.3 × 0.3 mm^2^) delivering ~10^11^ photons/second flux.

For measurements, we employed a 4-circle Huber diffractometer (Huber Diffraktionstechnik GmbH, Rimsting, Germany) (2θ precision <0.0001°) equipped with high-count-rate scintillator detector (0D detection scheme).

Painted samples were analyzed intact in reflection geometry using a flat sample holder (Ø 100 mm) mounted on precision xyz motor stage (1 μm resolution) with 360° φ-rotation and ±90° χ-tilting capabilities, enabling analysis of heterogeneous layers without mechanical separation.

Wood samples were finely ground and analyzed in transmission mode using a rotating capillary holder to ensure powder averaging.

Diffraction patterns were collected over 2θ = 4–70° with a 0.01° step size and 1 s exposure per point (~110 min total acquisition). Instrumental calibration was performed using the NIST LaB_6_ standard (SRM 660c) for wavelength and zero-shift correction. Data were processed using PDF4+ software (ICDD) for phase identification and quantification [19]. https://www.icdd.com/pdf-4-minerals/, accessed on 12 November 2025.

The 4–30° range was used to determine cellulose CrI, and angles above 30° were analyzed for pigment crystalline phases. Data were processed using PDF4 software (ICDD).

Quantitative phase analysis was performed using Rietveld refinement in PANalytical HighScore Plus software. (Malvern Panalytical, Almelo, Netherlands, https://www.malvernpanalytical.com/, accessed on 1 December 2025) Crystal structures were sourced from the ICDD PDF-4+ database. Refinement parameters included scale factors, lattice parameters, zero-shift correction, pseudo-Voigt peak profile functions, and March–Dollase preferred orientation corrections. Phase weight percentages were calculated from refined scale factors with estimated standard deviations (ESDs) derived from refinement statistics. Refinement quality was assessed using goodness-of-fit (GoF) and weighted R-profile (Rwp) indicators; all refinements achieved GoF < 2.5 and Rwp < 15%.

The degree of wood degradation was quantified through cellulose crystallinity measurements employing the Segal method, a widely adopted peak height approach. The crystallinity index (CrI%) was calculated according to Equation (1) [9,10,19]:

CrI% = [(I_200_ − Iam)/I_200_] × 100 (1)

where I_200_ represents the maximum intensity of the (200) reflection (2θ ≈ 15.0°), corresponding to the crystalline cellulose component, and Iam denotes the intensity minimum between the (200) and (110) reflections (2θ ≈ 12.5°), attributed to the amorphous cellulose fraction [19].

#### 2.3.3. Fourier Transform Infrared Spectroscopy

Molecular characterization of the pigment samples was performed using transmission mode on a Bruker VERTEX 80 FTIR Vertex 80 FTIR spectrometer from Bruker Optik GmbH, Ettlingen, Germany, following the KBr pellet method. Spectra were collected across the mid-infrared range from 4000 to 400 cm^−1^. All measurements were conducted at a spectral resolution of 4 cm^−1^ to resolve fine spectral features. To maximize the signal-to-noise ratio, each presented spectrum is the average of 100 consecutive scans. Data acquisition and initial processing, including atmospheric correction, were performed using Bruker OPUS software (version 8.0).

FTIR spectroscopy is a powerful tool for assessing the chemical state and degradation of archeological wood. Quantitative evaluation uses spectral indices: the lignin/carbohydrate ratio (peaks ~1505 and 1370 cm^−1^) indicates polysaccharide degradation, the carbonyl index (~1630 cm^−1^) reflects oxidation of lignin and hemicelluloses, and the crystallinity index (~1370 and 2900 cm^−1^) provides insight into cellulose structural order [19,30,31].

#### 2.3.4. Confocal Optical Microscopy

The surface morphology and micro-topography of the collected samples were investigated using a WITec Alpha300 R confocal microscope (WITec GmbH, Ulm, Germany). This system provides high-resolution optical sectioning, enabling the non-destructive characterization of particle size, distribution, and surface features. Samples were placed on a glass slide and analyzed without further preparation to preserve their original state. The microscope was operated in reflection mode and high-resolution images were acquired [32].

## 3. Results and Discussion

### 3.1. Wood Identification

**Figure 2:**
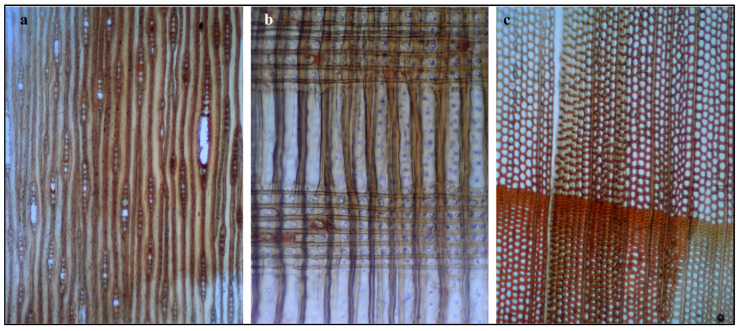
Light microscopy images of archeological wood sections: (**a**) tangential longitudinal section (TLS); (**b**) radial longitudinal section (RLS); and (**c**) transverse section (TS). Key features include diffuse-porous vessels, vasicentric paratracheal parenchyma (lighter zones around vessels in (**c**), uniseriate to biseriate rays (**a**,**b**), and heartwood–sapwood differentiation (**c**). These characteristics identify the wood as *Vachellia nilotica* (Fabaceae), commonly used in Late Period Egyptian artifacts. Images at 100× magnification using bright-field transmitted light microscopy.

Anatomical characterization of the archeological wood sample was performed through light microscopy examination of three principal anatomical sections: tangential longitudinal (Figure 2a), radial longitudinal (Figure 2b), and transverse (Figure 2c). This comprehensive three-plane analysis is essential for accurate species identification according to IAWA (International Association of Wood Anatomists) standardized protocols for hardwood identification [33].

Tangential Longitudinal Section (Figure 2a): The tangential section shows longitudinally aligned vascular elements and axial parenchyma, with vessels arranged in a diffuse-porous pattern. Elongated vessels with visible perforation plates indicate a hardwood species. Spindle-shaped rays, predominantly uniseriate to biseriate, provide diagnostic features consistent with Acacia species [6,33,34].

Radial Longitudinal Section (Figure 2b): The radial section reveals horizontal bands of parenchyma cells intersecting vertically oriented vessels and fibers, with rectangular to procumbent ray cells extending across multiple growth layers. Vessel-ray pitting appears of intermediate size. The observed horizontal banding and cellular arrangement are characteristic of the radial anatomy of Fabaceae (Leguminosae), consistent with *Vachellia nilotica* [6,33,34,35].

Transverse Section (Figure 2c): The cross-section provides key diagnostic features for species identification, showing a diffuse-porous structure with uniformly distributed circular to oval vessels arranged in a honeycomb pattern and high vessel frequency. Paratracheal parenchyma forms vasicentric sheaths around vessels, a diagnostic trait of Vachellia species. Color differentiation indicates the sapwood–heartwood transition, with the deep orange-brown heartwood characteristic of *Vachellia nilotica* [6,33,34,35,36].

Based on the combined microscopic evidence from all three anatomical planes, the wood sample is identified as *Vachellia nilotica* (L.) P.J.H. Hurter & Mabb. (synonymous with *Acacia nilotica* (L.) Willd. ex Delile), commonly known as Egyptian thorn [6,9,37].

*Vachellia nilotica* is a dense hardwood characterized by a relatively high lignin content and moderate to high natural durability, properties that generally confer resistance to biological degradation [38]. However, prolonged exposure to burial conditions, environmental stress, and microbial activity can overcome these intrinsic protective features, leading to structural and chemical deterioration over archeological timescales [39]. The observed degradation patterns therefore reflect the combined effects of wood anatomy, inherent material properties, and long-term environmental influence rather than species susceptibility alone [40].

The identification of *Vachellia nilotica* is consistent with extensive archaeobotanical evidence from ancient Egypt. This species represents one of the most abundantly utilized indigenous timber resources throughout ancient Egyptian history, documented from predynastic periods through the Late Period and Ptolemaic era [6,9].

### 3.2. Assessment of Wood Degradation

**Figure 3:**
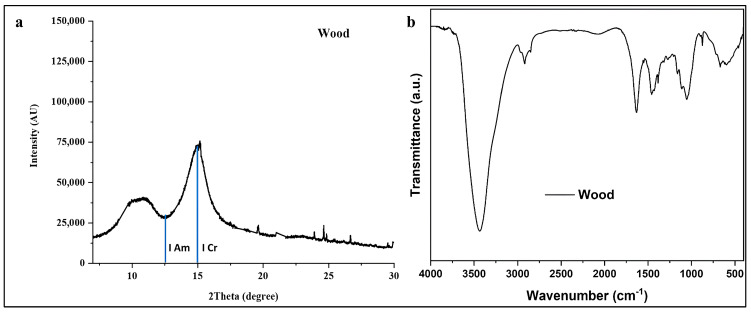
(**a**) XRD pattern and (**b**) FTIR spectrum of archeological wood for degradation assessment. XRD shows crystalline and amorphous cellulose peaks with CrI = 62.9%. FTIR displays lignocellulosic bands at 3435 cm^−1^ (O-H), 2919 cm^−1^ (C-H), 1630 cm^−1^ (C=O), 1510 cm^−1^ (aromatic lignin), and 1200–900 cm^−1^ (cellulose). Results indicate moderate degradation with preserved crystalline cellulose and lignin structures.

The X-ray diffraction pattern of the archeological wood sample (Figure 3a) exhibits characteristic cellulose patterns, providing quantitative insight into the degree of structural degradation. The diffractogram displays two prominent peaks corresponding to the crystallographic planes of cellulose I, the native form of cellulose in wood: the amorphous region (Iam) at approximately 2θ = 12.5° and the principal crystalline peak (I200) at 2θ ≈ 15.0°, corresponding to the (200) lattice plane of cellulose Iβ [19].

The relative intensity and sharpness of the (200) reflection serve as critical indicators of cellulose crystallinity preservation [9,19]. The observed peak profile demonstrates a well-defined crystalline peak with moderate intensity, suggesting partial retention of the original cellulose crystalline structure despite prolonged burial conditions. The presence of a distinct amorphous minimum (Iam) at approximately 2θ = 12.5° between the two main reflections indicates that both crystalline and amorphous cellulose domains persist in the degraded wood matrix [8,41,42].

Application of the Segal method yielded a crystallinity index (CrI) of 62.9%, which can be interpreted within the context of archeological wood degradation. Fresh, undegraded *Vachellia nilotica* wood typically exhibits CrI values ranging from 65 to 67.2%, depending on environmental and genetic factors [9]. The measured CrI value indicates moderate degradation with significant preservation of crystalline cellulose domains. This finding suggests that while the wood has undergone partial depolymerization and loss of amorphous components over its burial period, the core crystalline cellulose structure has maintained structural integrity sufficient for artifact preservation [19].

The background intensity and peak widths offer insights into the disorder of cellulose microfibrils. The relatively low background across 2θ = 4–30° indicates minimal amorphous or degraded organic matter, suggesting that neither extensive mineralization nor complete cellulose decomposition have not occurred [1,7,8,12,19]. However, moderate peak broadening, relative to fresh wood reported in the literature (based on full-width at half-maximum analysis), indicates reduced crystallite size and/or increased lattice strain, consistent with hydrolytic and oxidative degradation occurring during long-term burial [7,8,12].

The FTIR spectrum (Figure 3b) provides complementary molecular-level information regarding the chemical composition and degradation state of the wood sample. The spectrum exhibits several diagnostic absorption bands characteristic of lignocellulosic materials, each providing specific information about the preservation state of wood components as reported in the literature [19,43,44,45,46,47,48]. The broad band at 3435 cm^−1^ corresponds to O–H stretching vibrations from cellulose hydroxyl groups and adsorbed water. Its intensity and width reflect cellulose’s hygroscopicity and hydrogen-bonding network. In degraded wood, changes in this band indicate altered moisture sorption and the disruption of hydrogen bonding due to polymer chain scission [7,8,43]. The sharp bands at 2919 cm^−1^ and 2852 cm^−1^ are attributed to asymmetric and symmetric C–H stretching of methyl and methylene groups, respectively, confirming the retention of aliphatic methylene groups in cellulose and hemicellulose structures [43,44,45,46,47,48]. The preservation of these bands indicates that the fundamental carbon backbone of polysaccharides remains largely intact, despite potential depolymerization [43,44,45,46,47,48]. The absorption at 1636 cm^−1^, attributed to H–O–H bending of adsorbed water and conjugated C=O stretching in hemicellulose acetyl groups and lignin carbonyl/carboxyl groups, shows reduced intensity compared to fresh wood spectra, indicating partial loss or alteration of hemicellulose [43,44,45,46,47,48]. Hemicellulose, as the most labile wood polymer, preferentially degrades during burial, reflected by the diminished intensity of carbonyl-associated bands [43,44,45,46,47,48]. This observation aligns with expected degradation patterns in archeological wood, where hemicellulose depletion occurs more rapidly than cellulose or lignin degradation [9,10,43]. A weak signal is detected at 1506 cm^−1^ which corresponds to the aromatic skeletal vibration of lignin [43,44,45,46,47,48]. The bands at 1457 cm^−1^ and 1384 cm^−1^ correspond to C–H deformation in lignin and carbohydrates. These bands provide information on lignin preservation [43,44,45,46,47,48,49,50]. Distinct aromatic bands indicate substantial preservation of the lignin matrix, which imparts structural rigidity and protects against microbial degradation. Lignin retention is crucial for archeological wood survival, as it shields cellulose microfibrils and limits enzymatic attack [9,10,19]. The band at 1318 cm^−1^ is attributed to Wagging of CH2 groups in crystalline cellulose, while the band at 1272 cm^−1^ is characteristic of aromatic C–O stretching vibrations of methoxyl and phenyl propane guaiacol ring units of lignin. These bands contain multiple diagnostic bands for cellulose structure and crystallinity [19]. The sharp absorptions at these bands reflect the integrity of the cellulose backbone. Notably, the bands at ~1160 cm^−1^ (C–O–C asymmetric bridge stretching) and ~1060 cm^−1^ (C–O stretching in cellulose and hemicellulose) remain well defined. Their preservation supports the XRD results, confirming that the cellulose polymer structure retains substantial integrity. Finally, the band at ~874 cm^−1^, corresponding to β-glycosidic linkages in cellulose, serves as a marker of crystallinity. Its presence and sharp profile corroborate the moderate cellulose crystallinity observed in XRD analysis [19]. Moreover, the FTIR spectrum of the wood substrate reveals a band at 1636 cm^−1^ and a weak feature at 1506 cm^−1^. To quantify the extent of oxidation, the carbonyl index was calculated from the ratio of these bands. The aromatic C=C stretch at 1506 cm^−1^ was selected as the internal reference because its vibrational mode remains relatively stable under the oxidative degradation pathways affecting lignin, unlike signals from more labile carbohydrate polymers. The index was calculated using the following equation:

$$\mathrm{Carbonyl}\;\mathrm{Index}\;=\;\frac{I_{1636}}{I_{1506}}$$

where ***I*_1636_** and ***I*_1506_** are the peak heights at the respective wavenumbers. The calculated carbonyl index value of 2.22 acts as a clear sign of oxidation, such that the increase in its value provides direct evidence of advanced degradation, showing that carbonyl groups have built up from the breakdown of the wood’s structural polymers [19,30,31].

The Total Crystallinity Index (***I*_1384_**/***I*_2919_**) was found to be 1.71. This index provides an estimate of the relative crystallinity of cellulose. It often increases in degraded wood because the more susceptible amorphous cellulose regions (represented by 2919 cm^−1^ band) are degraded first, leaving behind a higher proportion of crystalline cellulose [19,30,31]. The most solid finding from these data is that the wood is moderately oxidized. The value of the carbonyl index and the changed shape of the lignin-related bands in the spectrum offer the clearest proof of the degree of damage [43,44,45,46,47,48,49,50].

Based on the XRD and FTIR indices, we conclude that the degree of degradation observed in the studied statuette is consistent with a moderate level of cellulose depolymerization when compared with other archeological wooden artifacts reported in the literature [8]. Highly degraded archeological woods typically exhibit a marked reduction in CrI and elevated carbonyl indices, whereas moderately preserved artifacts retain partial cellulose crystallinity and show limited oxidation [51].

The moderate structural preservation observed can be partly attributed to the intrinsic characteristics of *Vachellia nilotica*, a dense hardwood with relatively high lignin content, which is known to enhance resistance to enzymatic and oxidative degradation [38]. Lignin-rich matrices act as protective barriers that delay microbial attack and cellulose depolymerization, thereby slowing long-term deterioration under burial conditions [52].

### 3.3. Characterization of Painted Colors

#### 3.3.1. Black Color

The XRF spectra for the black pigments are dominated by calcium, which is likely due to the calcite preparation layer beneath the paint. The absence of heavy metals like manganese is notable, and combined with the presence of a carbon-based black, it is likely that the pigment is carbon black (soot or charcoal) [53]. The presence of silicon is likely due to fine silicate sand or quartz particles, either naturally present as an impurity in the charcoal/soot or intentionally added as an extender.

**Figure 4:**
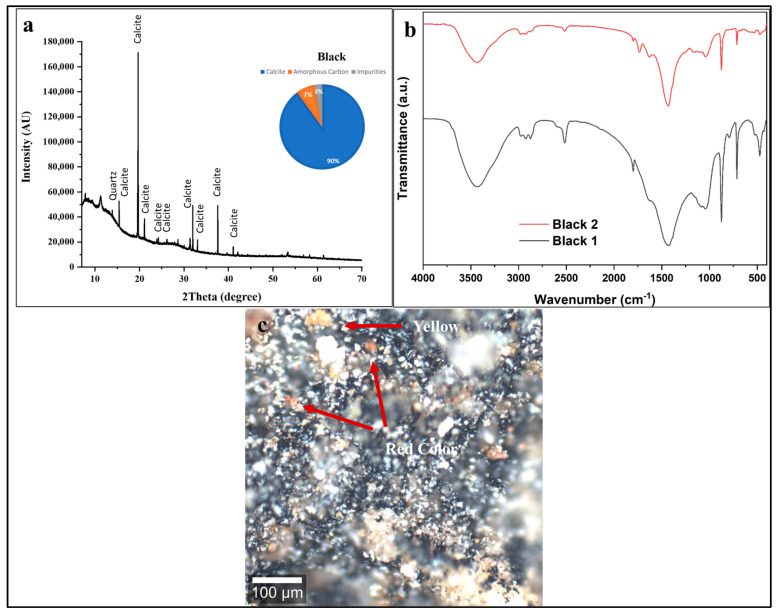
(**a**) XRD pattern, (**b**) FTIR spectra, and (**c**) confocal microscopy of black decorative layer. XRD identifies calcite ground (90 ± 2 wt %) with carbon black and quartz. FTIR shows carbonate bands (1420, 870 cm^−1^), degraded organic binder (2900 cm^−1^), and spatial variation. Confocal microscopy reveals heterogeneous distribution with black carbon particles and red ochre inclusions (red arrows, indicating intentional color modification), plus degradation features: cracking, fragmentation, and detachment. Scale bar = 100 μm.

In Figure 4a, the XRD pattern of the black decorative layer reveals a distinctive crystalline profile dominated by an exceptionally intense reflection corresponding to calcite (CaCO_3_) at 2θ ≈ 19.4°, with additional characteristic calcite peaks at 2θ ≈ 15.4°, 21°, 32°, 38°, 41°, and 53° [54]. The overwhelming predominance of calcite reflections, as illustrated in the pie chart (showing approximately 90 ± 2 wt % calcite content), indicates that calcium carbonate serves as the primary white filler or substrate matrix within the black decorative layer. The presence of minor phases identified as quartz (SiO_2_) reveals the multi-component nature of this decorative coating. Importantly, no crystalline black phases were detected in the diffractogram, supporting the interpretation that the black coloration is most likely attributable to amorphous carbon [55]. The relatively low background intensity and the absence of a broad amorphous hump in the 2θ = 15–30° range suggests minimal organic binder content or significant degradation of original organic components over approximately 2600 years of burial [54].

The XRD pattern is consistent with the use of carbon-based black pigment (charcoal or soot) mixed with a calcium carbonate-rich preparation layer or ground. This compositional profile aligns with well-documented ancient Egyptian pigment technology [2,54,55].

This pigment formulation is characteristic of Saite Period (26th Dynasty) artistic practices, where calcium carbonate-based grounds were routinely applied to wooden statuary before polychrome decoration, following traditions established in earlier pharaonic periods [2,54].

The FTIR spectra of two sampling positions (Black 1 and Black 2) in Figure 4b reveal both consistency and subtle variations in the chemical composition of the black layer, providing insights into the organic binder system and degradation state. The lack of any distinct vibrational bands for crystalline pigments like manganese oxides, which would appear between 500 and 600 cm^−1^ [2,54,55,56,57], supports the identification of the pigment as an amorphous carbon such as charcoal or soot [56,57].

The intense, broad O-H stretching absorption band centered around 3400 cm^−1^ indicates the presence of hydroxyl groups from absorbed atmospheric moisture in the porous calcite/carbon matrix, residual organic binder, or possible degradation products. Weak but discernible absorption bands in the 2960–2850 cm^−1^ region (aliphatic C–H stretching) suggest the presence of residual organic material. While such signals can be associated with degraded binding media, including plant gums, they are not diagnostic for a specific binder type in this context. The absence of discernible protein amide bands (~1640, ~1540 cm^−1^) indicates that a proteinaceous binder such as egg yolk or animal glue is not a major component; however, the FTIR data alone cannot conclusively identify the original organic medium due to signal overlap from degradation products and the complex mineral–organic matrix. Carbonate Absorption Bands (1500–1400 cm^−1^ and 870 cm^−1^): The strong, sharp absorption around 1420 cm^−1^ (asymmetric CO_3_^2−^ stretching) and 870 cm^−1^ (out-of-plane CO_3_^2−^ bending) confirms the dominant presence of calcite (CaCO_3_), corroborating the XRD findings. The characteristic calcite doublet around 2500 cm^−1^ (combination bands) is also visible [2,54,55,56,57].

Silicate Region (1200–900 cm^−1^): Absorption features in this region, particularly around 1080 cm^−1^, indicate Si-O stretching vibrations from quartz or clay minerals, consistent with the minor quartz phase detected by XRD [2,54,55,56,57].

Characteristic FTIR absorption bands indicative of a carbon-based black pigment are observed at ~1580–1600 cm^−1^, corresponding to C=C stretching vibrations of aromatic or graphitic carbon, at ~1400 cm^−1^ related to C–H bending modes, and at ~2850–2920 cm^−1^ assigned to aliphatic C–H stretching from residual organic components, consistent with the use of charcoal or soot [56,57].

The Black 2 spectrum shows overall higher absorption intensity than Black 1, particularly in the hydroxyl, aliphatic, and carbonate regions, indicating local variations in moisture content, organic binder preservation, and calcite ground layer thickness. These differences reflect spatial heterogeneity typical of hand-applied ancient pigment layers with variable binder-to-pigment ratios. The absence of amide I and II bands further suggests the use of a plant-based rather than proteinaceous binder [58,59].

The confocal microscopy image (Figure 4c) reveals a heterogeneous, particulate black layer dominated by dark carbon-based pigment particles (charcoal or soot). The irregular particle sizes and agglomerated morphology are characteristic of traditionally prepared carbon pigments produced by incomplete combustion, with particles ranging from submicron to tens of micrometers [60].

The red/orange particles (red arrows) likely correspond to red ochre (hematite, Fe_2_O_3_) intentionally mixed with the black pigment to modify the color and achieve specific esthetic or symbolic effects [60,61]. The extensive microscale cracking and fragmentation observed indicate advanced binder degradation, loss of cohesion, and mechanical stress related to wood dimensional changes during burial and post-excavation, leading to embrittlement of the original organic binder system [60,61].

#### 3.3.2. Blue Color

The copper signal in the XRF spectra of blue pigments is a clear indicator of the synthetic pigment Egyptian Blue (cuprorivaite, CaCuSi_4_O_10_) [62]. The consistent presence of calcium and silicon in the spectra further supports this identification, as these elements are fundamental constituents of the cuprorivaite crystal structure [63]. The trace amounts of iron are not a component of Egyptian Blue and might be due to mineral impurities in the quartz sand used as a raw material in the synthesis of Egyptian Blue [64].

**Figure 5:**
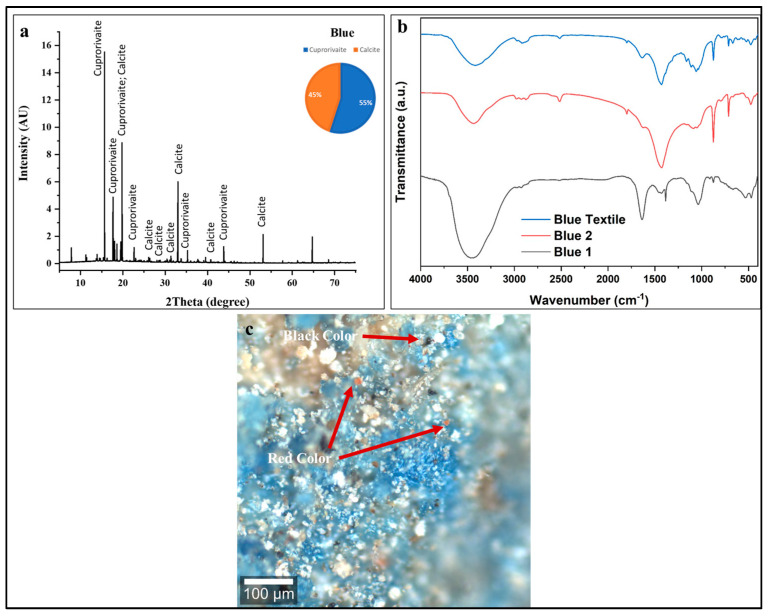
(**a**) XRD pattern, (**b**) FTIR spectra, and (**c**) confocal microscopy of blue decorative layer showing Egyptian Blue (cuprorivaite, 55 ± 5 wt %) with calcite (45 ± 2 wt %). XRD confirms synthetic CaCuSi_4_O_10_ with characteristic reflections. FTIR shows Egyptian Blue silicate bands (1000–1100 cm^−1^), calcite carbonates (1420, 870 cm^−1^), and minimal organic binder. Confocal microscopy reveals intentional blue–black pigment mixture (red arrows indicate carbon black additions for color darkening) with severe degradation: binder loss, particle detachment, surface fragmentation, and material discontinuity requiring conservation treatment. Scale bar = 100 μm.

The X-ray diffraction pattern of the blue decorative layer (Figure 5a) reveals a distinctive crystallographic signature dominated by cuprorivaite (CaCuSi_4_O_10_), the synthetic copper–calcium silicate pigment universally known as Egyptian Blue [54,55,56,57,58,59]. Pie chart quantification reveals cuprorivaite (Egyptian Blue) at ~55 ± 5 wt %—the primary chromophore and intentionally synthesized pigment phase. Calcite (CaCO_3_) at 45 ± 2 wt % is the secondary phase serving as the preparation layer. This compositional ratio is highly significant and indicates a sophisticated manufacturing approach rather than simple pigment application [54,55,56,57,58,59].

In Figure 5b, the FTIR spectra from three sampling positions (Blue, Blue 1, Blue 2) provide complementary molecular-level information about the blue decorative layer’s composition, confirming and expanding upon the XRD findings. The spectra of blue pigment indicate significant degradation of the Egyptian Blue structure. The diagnostic, strong Si-O-Si asymmetric stretching vibrations, typically a sharp doublet between 1000 and 1200 cm^−1^ [63], are severely diminished. The presence of a weak, residual Si-O-Si symmetrical stretching mode identified via the KBr method near 665 cm^−1^ confirms the pigment’s identity but also its advanced structural deterioration [63].

The deterioration of the Egyptian blue pigment, as indicated by its FTIR spectral features, is attributed to chlorination, a conclusion supported by the detection of chlorine via XRF in both the blue and green pigments and definitively confirmed by the identification of atacamite in the green pigment through XRD analysis [63,65,66]. All three spectra show this prominent silicate feature, confirming Egyptian Blue as the primary blue chromophore. Strong absorption around 1420 cm^−1^ (CO_3_^2−^ asymmetric stretch) confirms significant calcite content, confirming the XRD results showing ~45% calcite. The intense, broad absorption centered around 3400 cm^−1^ indicates atmospheric moisture in the porous pigment/calcite matrix, with the bandwidth and intensity suggesting a significant hygroscopic nature [54,55,56,57,58,59,60]. Similar to analysis of the black layer, the FTIR spectra lack intense, well-defined amide bands.

The confocal microscopy image, (Figure 5c) shows a heterogeneous, multicolored surface with distinct regions. The pigment appears as aggregated crystalline particles ranging from fine (few microns) to coarse (tens of microns) grain sizes [60,61]. Particle morphology shows angular, fractured crystalline forms typical of ground synthetic Egyptian Blue. The color intensity varies across the field, indicating non-uniform pigment concentration and particle size distribution. Black regions appear as discrete particles and larger agglomerated zones distributed throughout the blue matrix. The spatial association between black and blue pigments indicates deliberate mixing rather than sequential application or contamination [60,61,67].

#### 3.3.3. Green Color

The strong copper signal in the spectrum of green pigment confirms that a copper-based green pigment was used. The common green pigment in this context is malachite (a basic copper carbonate, Cu_2_CO_3_(OH)_2_) [63,65]. The presence of chlorine is particularly noteworthy, as it may suggest the formation of atacamite (Cu_2_Cl(OH)_3_) [66]. Malachite can deteriorate into atacamite through processes involving copper corrosion, particularly in the presence of chloride ions from burial environments [66,67]. XRF analysis also revealed that the pigment contains silicon, aluminum, and potassium, indicating the presence of a siliceous substrate mixed with the pigments [1].

**Figure 6:**
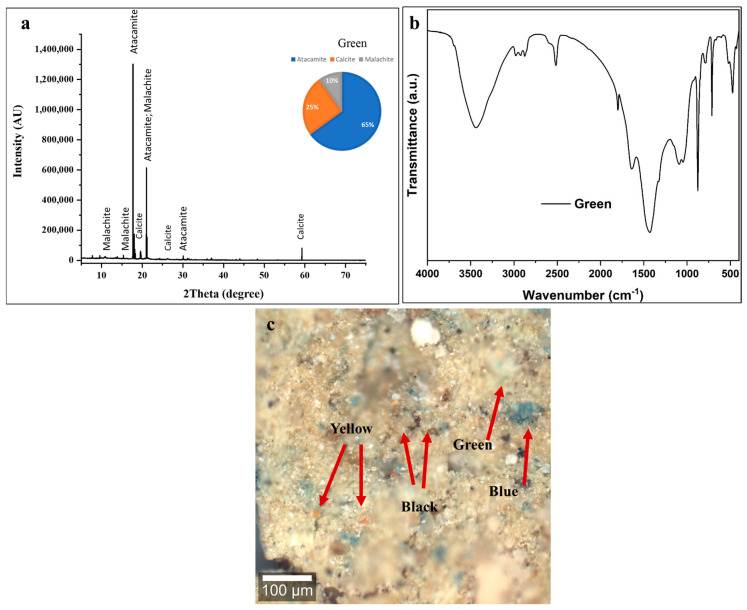
(**a**) XRD pattern, (**b**) FTIR spectrum, and (**c**) confocal microscopy of green layer showing atacamite-dominated composition (65 ± 5 wt % Cu_2_Cl(OH)_3_, 10 ± 2 wt % malachite, 25 ± 2 wt % calcite). High atacamite indicates chloride-driven alteration from original malachite. FTIR confirms copper minerals (3400, 1420, 870 cm^−1^) with minimal organic binder. Confocal microscopy shows intentional multi-pigment mixing: green copper minerals with Egyptian Blue, yellow ochre, and carbon black (arrows) for color adjustment. Critical degradation: complete binder failure, fragmentation, material loss, and discontinuous coverage requiring conservation. Scale bar = 100 μm.

In Figure 6a, the diffractogram exhibits characteristic reflections corresponding to three distinct crystalline phases, with quantitative phase analysis indicating atacamite (Cu_2_Cl(OH)_3_) as the dominant component at approximately 65 ± 5 wt %, accompanied by calcite (CaCO_3_) at 25 ± 2 wt %, and malachite (Cu_2_CO_3_(OH)_2_) at 10 ± 2 wt % of the total crystalline composition [54,55]. The predominance of atacamite, a copper chloride hydroxide mineral, represents a particularly significant finding in the context of ancient Egyptian green pigment usage. This copper-based green mineral forms through specific geochemical conditions involving the interaction of copper compounds with chloride-bearing solutions, and its dominance in this paint layer raises important questions regarding original pigment composition versus diagenetic alteration during the 2600-year burial period [65,66,67]. The presence of malachite, though constituting only 10 ± 2 wt % of the crystalline phases, is archeologically significant as this natural copper carbonate mineral (Cu_2_CO_3_(OH)_2_) represents one of the most traditional and widely used green pigments throughout pharaonic Egyptian history. The substantial calcite content (25 ± 2 wt %) follows the pattern observed in both the black and blue decorative layers, confirming the systematic application of calcium carbonate-based ground layers [53,54,55]. Alternatively, and perhaps more plausibly given the burial context, the high atacamite content may reflect post-depositional transformation of originally malachite-dominated green pigment through reaction with chloride-bearing groundwater or salt deposits within the tomb environment [65,66,67]. Egyptian burial contexts frequently contain elevated chloride concentrations from natron used in mummification, natural evaporite deposits, or infiltrating saline groundwater. Over millennia, malachite can undergo chloride-mediated transformation according to the reaction Cu_2_CO_3_(OH)_2_ + Cl^−^ → Cu_2_Cl(OH)_3_ + CO_3_^2−^, progressively converting the original copper carbonate pigment to copper chloride hydroxide while maintaining the green coloration but altering the mineralogical composition [65,66,67]. This diagenetic process, well documented in archeological copper-based pigments from various contexts, would explain the unusual dominance of atacamite over malachite in this sample [65,66,67].

In Figure 6b, the FTIR spectrum exhibits several diagnostic absorption features that collectively confirm the mixed copper-based green pigment composition with substantial calcite content and minimal preserved organic binder [54,55,56,57]. The broad, intense absorption band centered around 3400 cm^−1^ corresponds to O-H stretching vibrations originating from multiple hydroxyl-bearing components within the paint layer. Both atacamite and malachite contain structural hydroxyl groups as integral components of their crystal structures, contributing to this absorption feature [65,66,67]. Additionally, adsorbed atmospheric moisture within the porous, degraded paint matrix and potential residual organic binder with hydroxyl functionalities further enhance this band. The bandwidth and intensity profile suggest significant water content and/or extensive hydrogen bonding networks characteristic of hydrated copper minerals [68,69]. Weak but discernible absorption bands in the 2920–2850 cm^−1^ region indicates the presence of aliphatic C-H stretching vibrations from trace organic materials [68,69]. The relatively low intensity of these bands, consistent with the black and blue layer analyses, confirms extensive degradation of the original organic-based binding medium over the burial period [70,71]. The degradation of any original organic binder is consistent with, and likely contributes to, the current friable and mechanically unstable condition of the decorative layer [71]. The strong absorption band at approximately 1420 cm^−1^, corresponding to asymmetric CO_3_^2−^ stretching vibrations, reflects contributions from both calcite in the ground layer and the carbonate groups present in residual malachite [53,54,55,56,57,58,59,60]. The companion band at 870 cm^−1^, representing out-of-plane CO_3_^2−^ bending, confirms the presence of the calcite polymorph specifically. The relative intensities of these carbonate features align with the XRD-determined phase proportions, indicating substantial calcite content [53]. The spectral region between 1200 and 900 cm^−1^ exhibits complex, overlapping absorption features arising from Cu-O vibrations in atacamite and malachite crystal structures, combined with calcite C-O stretching modes [66,67]. The copper–oxygen bonds in both copper chloride hydroxide (atacamite) and copper carbonate hydroxide (malachite) produce characteristic mid-infrared absorptions in this region, though precise band assignment is complicated by spectral overlap with calcite features [53,60,66,67]. The FTIR spectrum confirms the XRD identification of a mixed copper-based green pigment system with dominant atacamite, subsidiary malachite and calcite, and extensively degraded organic binding medium.

The confocal microscopy image (Figure 6c) reveals a heterogeneous, multicolored surface composed of green, blue, yellow, black, and light-colored particles in close spatial association, indicating intentional mixing of multiple pigments to achieve complex color effects [60,61]. The green particles, corresponding to copper-based pigments (atacamite and residual malachite) identified by XRD and FTIR, occur as irregular crystalline fragments with particle sizes ranging from a few micrometers to several tens of micrometers, consistent with traditionally ground mineral pigments [65,66]. Their blue-green to emerald hues under reflected light vary with particle size, crystal orientation, and the degree of surface alteration [61].

The observed multi-pigment complexity is best explained by intentional mixing of green, blue, yellow, and black pigments by ancient artisans to produce specific, nuanced color effects suited to particular iconographic elements. This practice reflects a sophisticated understanding of subtractive color mixing and highlights the advanced technical capabilities of Late Period Egyptian painters [72,73].

#### 3.3.4. Yellow Color

XRF analysis of the yellow pigment shows strong arsenic peaks, confirming the presence of orpiment (As_2_S_3_) as the primary yellow chromophore. Dominant calcium peaks reflect substantial calcite (~40 ± 2 wt %) from the underlying preparation layer and/or pigment filler. A minor iron peak indicates either natural contamination from iron oxide impurities in orpiment (yellow ochre) or intentional admixture of red iron oxide to modify the hue [72,73].

**Figure 7:**
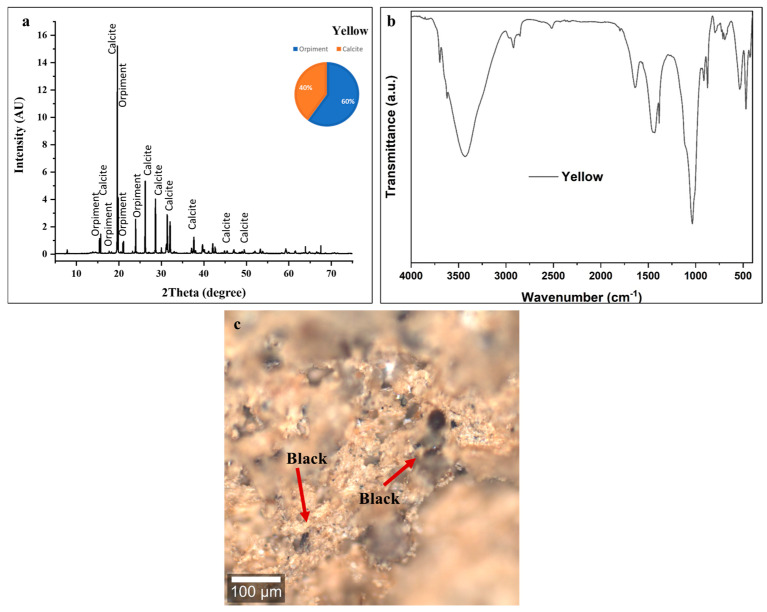
Compositional and microstructural analysis of yellow layer. (**a**) XRD identifies orpiment (As_2_S_3_, 60 ± 5 wt %) and calcite (40 ± 2 wt %); (**b**) FTIR shows hydroxyl groups (3400 cm^−1^), trace degraded binder (2900 cm^−1^), calcite carbonates (1420, 870 cm^−1^), and As-S vibrations (<1200 cm^−1^). (**c**) Confocal microscopy reveals intentional yellow–black pigment mixture: orpiment systematically mixed with carbon black (arrows) for chromatic modification, creating warmer earth-toned yellows and extending expensive arsenic sulfide pigment. Scale bar = 100 μm.

The XRD pattern in Figure 7a shows characteristic orpiment (As_2_S_3_) reflections at multiple 2θ positions, with the most prominent peaks appearing around 15.2°, 17.1°, 20°, 21°, and 24° [62]. Phase quantification analysis indicates that orpiment constitutes approximately 60 ± 5 wt % of the crystalline material, with calcite (CaCO_3_) comprising the remaining 40 ± 2 wt %. This substantial calcite content follows the consistent pattern observed across all colored decorative layers on this statue, confirming the systematic application of calcium carbonate-based ground layers as the standard preparatory technique [53,54,55,56,57,58,59,60,61].

The FTIR spectrum shown in Figure 7b of the yellow layer provides molecular-level confirmation of the orpiment and calcite composition identified through XRD analysis.

The broad absorption band centered around 3400 cm^−1^ corresponds to O-H stretching vibrations from hydroxyl groups in adsorbed atmospheric moisture and residual organic binder components. The moderate intensity of this feature indicates the significant hygroscopic nature of the porous paint matrix, with water molecules associated with the calcite and potentially with degraded organic materials [53,54,55,56,57,58,59,60,61].

Weak absorption features in the 2920–2850 cm^−1^ region indicate trace aliphatic C-H stretching vibrations from residual organic binding medium. As observed in all other colored layers on this statue, the low intensity of these organic binder signatures reflects near-complete degradation of the original organic binder that bound pigment particles together and adhered them to the substrate. Minimal organic material remains, contributing to the mechanically unstable, friable condition of the paint layer [53,54,55,56,57,58,59,60,61].

The strong, sharp absorption bands at 1420 cm^−1^ (asymmetric CO_3_^2−^ stretching) and 870 cm^−1^ (out-of-plane CO_3_^2−^ bending) provide unambiguous identification of calcite as a major component of the yellow decorative layer. These characteristic carbonate vibrations confirm the substantial (40 ± 2 wt %) calcite content determined through XRD quantification, representing both the preparation ground layer and possible intentional calcite addition as a pigment extender [53,54,55,56,57,58,59,60,61].

The broad, complex absorption features extending from approximately 1200 cm^−1^ down to the lower wavenumber limit of the spectrum reflect As-S stretching and bending vibrations characteristic of the orpiment (As_2_S_3_) crystal structure [53,54,55,56,57,58,59,60,61]. The exceptionally strong absorption centered around 1000 cm^−1^ dominates the spectrum and likely represents overlapping contributions from both calcite (C-O stretching modes) and potential silicate impurities. This intense feature extends across a wide wavenumber range (approximately 1200–900 cm^−1^), creating the broad absorption valley characteristic of complex mineral mixtures containing multiple oxide and sulfide phases [53,54,55,56,57,58,59,60,61].

Characteristic FTIR bands at ~1637 cm^−1^ attributed to C=C stretching of aromatic or graphitic carbon, at ~1430 cm^−1^ corresponding to C–H bending vibrations, and at ~2927 cm^−1^ associated with aliphatic C–H stretching from residual organic matter are consistent with the presence of carbon-based impurities [73].

The confocal microscopy image (Figure 7c) shows a heterogeneous surface exhibiting yellow orpiment pigment particles intermixed with substantial quantities of black carbon-based pigment, creating a complex, multicolored appearance rather than the pure yellow tone expected from the macroscopic layer designation.

The yellow particles, representing orpiment (As_2_S_3_) crystals, appear as irregularly shaped fragments with characteristic golden-yellow to orange-yellow coloration under reflected light. The particle size distribution shows considerable variability, ranging from fine particles to relatively coarse fragments, typical of traditionally ground mineral pigments [65].

The presence of abundant black pigment particles (indicated by arrows) intimately mixed with the yellow orpiment represents, most plausibly, the intentional mixing of yellow orpiment with carbon black by ancient Egyptian craftsmen to create modified yellow tones ranging from golden-brown to ochre or khaki colors [72,73].

The microscopy image provides compelling evidence of severe degradation affecting the yellow decorative layer. The paint surface exhibits a completely fragmented, particulate appearance with no evidence of continuous coating integrity. Individual pigment particles appear loosely aggregated with extensive gaps and voids between clusters, indicating complete loss of the organic binder matrix that originally held particles in cohesive adhesion [72,73].

#### 3.3.5. Red Color

The high intensity of iron in the spectra is a definitive indicator of the use of an iron oxide-based pigment [74]. This identifies the material as red ochre, a naturally occurring earth pigment whose primary coloring agent is hematite (Fe_2_O_3_) [54,63,74]. The strong iron signal dominates the spectrum, confirming its role as the intended colorant. The presence of aluminum and silicon is intrinsic and expected in red ochre, confirming the use of a natural, unrefined earth pigment [63]. The presence of chlorine and sulfur may be due to soluble salts that have migrated into the porous paint layer from the burial environment.

**Figure 8:**
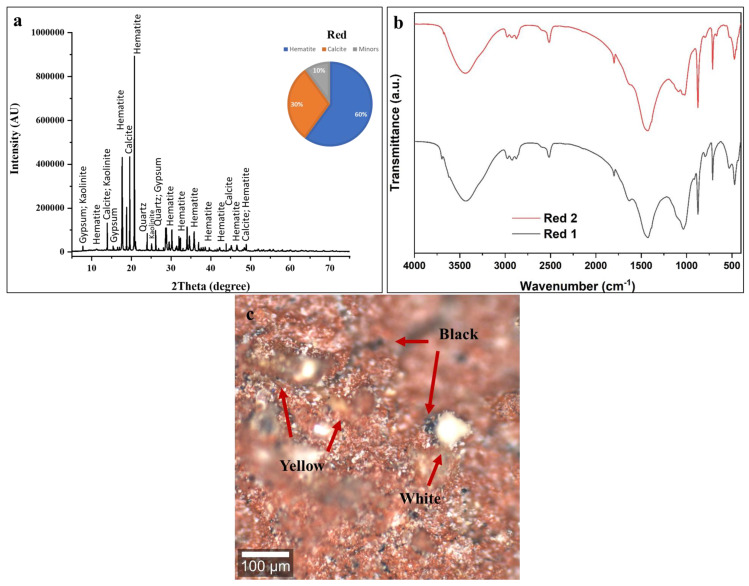
Compositional and microstructural analysis of red layer. (**a**) XRD identifies hematite (Fe_2_O_3_, 60 ± 5 wt %), calcite (30 ± 2 wt %), and trace alunite (10 ± 2 wt %); (**b**) FTIR from two positions (Red 1, Red 2) shows hydroxyl groups (3400 cm^−1^), trace degraded binder (2900 cm^−1^), calcite carbonates (1420, 870 cm^−1^), and Fe-O vibrations (<1000 cm^−1^); spectral variations indicate spatial heterogeneity in pigment-to-calcite ratio. (**c**) Confocal microscopy reveals intentional multi-pigment formulation: red hematite mixed with carbon black (arrows) for darker burgundy/brown-red tones, plus yellow ochre and white calcite. Scale bar = 100 μm.

The XRD pattern in Figure 8a exhibits numerous crystalline reflections corresponding to three principal mineral phases: hematite (Fe_2_O_3_) at approximately 60 ± 5 wt %, calcite (CaCO_3_) at 30 ± 2 wt %, and minor quantities of Kaolinite (KAl_3_(SO_4_)_2_(OH)_6_) at roughly 10 ± 2 wt %. Hematite, the dominant chromophore responsible for red coloration, displays its characteristic reflections corresponding to the rhombohedral crystal structure of this iron (III) oxide mineral [57,58,74]. The substantial calcite content (30 ± 2 wt %) continues the pattern observed throughout all colored layers on this statue, confirming the systematic application of calcium carbonate ground layers beneath pigmented coatings [54,55,56,57,58,59,60]. The detection of trace Kaolinite reflects several possibilities: natural occurrence as an impurity within the red ochre mineral source, intentional addition as a mordant or fixative agent to improve pigment adhesion or color stability; or post-depositional formation through reaction between burial environment sulfates and aluminum-bearing components in the paint or wood substrate. Given the minor concentration, its presence most likely represents a natural mineral impurity within the processed red ochre [57].

The FTIR spectra shown in Figure 8b from two sampling positions (Red 1 and Red 2) exhibit similar absorption band patterns, indicating relatively homogeneous composition across the red decorated surface, though minor intensity variations reflect spatial heterogeneity in pigment-to-calcite ratios and binder distribution typical of hand-applied ancient coatings [53,54,55,56,57,58,59,60]. The broad absorption band centered around 3400 cm^−1^ corresponds to O-H stretching vibrations from multiple hydroxyl-bearing sources including adsorbed atmospheric moisture in the porous paint matrix, structural hydroxyl groups in Kaolinite (which contains OH^−^ in its crystal structure), and residual organic binder components. The bandwidth and intensity suggest significant water content associated with the hygroscopic iron oxide and calcite minerals [53,54,55,56,57,58,59,60]. Weak absorption features in the 2920–2850 cm^−1^ region indicate trace aliphatic C-H stretching vibrations from residual organic binding medium. Consistent with all other analyzed colored layers, the minimal intensity of these organic signatures confirms near-complete degradation of the original organic binder [53,54,55,56,57,58,59,60]. The strong absorption bands at 1420 cm^−1^ (asymmetric CO_3_^2−^ stretching) and 870 cm^−1^ (out-of-plane CO_3_^2−^ bending) provide diagnostic identification of calcite, confirming the substantial (30 ± 2 wt %) calcium carbonate content determined through XRD quantification. The spectral region below 1000 cm^−1^ exhibits complex absorption features attributable to Fe-O vibrations from the hematite crystal structure. Iron oxides produce characteristic infrared absorption patterns in this lower wavenumber region, though precise band assignments are complicated by the polymeric nature of the Fe-O bonding network and potential overlap with calcite and alunite features [55,56,57,58]. The moderate absorption features around 1100–1200 cm^−1^ may reflect SO_4_^2−^ stretching vibrations from the trace Kaolinite component (KAl_3_(SO_4_)_2_(OH)_6_), which contains sulfate groups as structural constituents [75]. The spectral variations between Red 1 and Red 2 sampling positions demonstrate compositional heterogeneity across the red decorated surface. Red 2 (upper trace) exhibits enhanced absorption intensity across most regions compared to Red 1 (lower trace), particularly in the hydroxyl (3400 cm^−1^) and carbonate (1420, 870 cm^−1^) bands. This suggests that the Red 2 sampling area contains a higher calcite concentration relative to hematite, a greater moisture content, or a thicker paint layer contributing more material to the analyzed volume.

The characteristic bands at ~1631 cm^−1^ (C=C stretching of aromatic/graphitic carbon), ~1427 cm^−1^ (C-H bending), and ~2921 cm^−1^ (aliphatic C-H stretching from organic residues) are consistent with the presence carbon-based impurities [72,73].

Confocal microscopy of the red decorative layer (Figure 8c) reveals notable compositional complexity, with a heterogeneous surface composed of red, black, yellow, and white particles in close spatial association rather than a uniform red coating. The red particles correspond to hematite (Fe_2_O_3_) and occur as irregularly shaped iron oxide fragments distributed across the microscopic field [72,73]. Under reflected light, the particles display characteristic red to reddish-brown hues, with variations in color intensity related to particle size. Notably, microscopy analysis reveals abundant black pigment particles intimately mixed with the red hematite. This deliberate combination of red iron oxide and black carbon is a well-documented ancient Egyptian painting practice used to produce darker, subdued tones ranging from burgundy to dark brown, suitable for shading, three-dimensional modeling, and specific iconographic requirements. Black carbon pigment was economically advantageous, being cheap and easy to produce through simple combustion, and served as an effective extender for more valuable iron oxide pigments without compromising color. The yellow particles seen in the microscopy images likely represent yellow ochre, naturally present as impurities in red ochre deposits, since natural red ochre often contains variable amounts of yellow iron oxyhydroxides, and ancient processing may not have fully separated these related minerals [72,73]. White/light areas show exposed calcite substrate from pigment loss or high calcite-to-pigment ratios. The distribution pattern suggests that ancient craftsmen intentionally mixed multiple pigments to create specific colors rather than using pure red [72,73].

#### 3.3.6. White and Golden Colors

**Figure 9:**
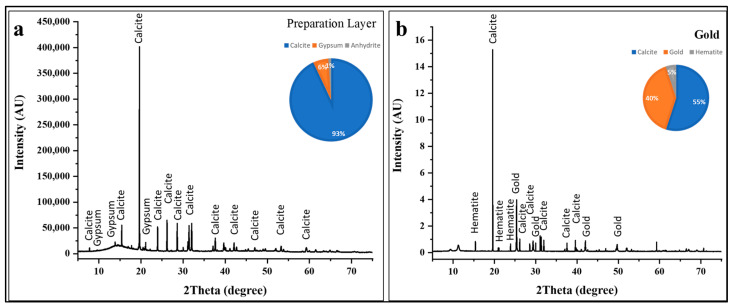
XRD analysis of substrate and metallic layers. (**a**) Preparation layer (gesso) showing dominant calcite (93 ± 5 wt %) with minor gypsum (6 ± 2 wt %) and anhydrite (1 ± 1 wt %). (**b**) Gold decoration revealing metallic gold (40 ± 2 wt %), underlying calcite preparation (55 ± 5 wt %), and hematite (5 ± 2 wt %); gold reflections confirm authentic ancient gilding using high-purity metallic gold applied as leaf or powder.

The XRD pattern of the base layer in Figure 9a exhibits an exceptionally intense calcite (CaCO_3_) reflection at 2θ ≈ 19.5°. Additional characteristic calcite reflections appear, providing unambiguous identification of this calcium carbonate polymorph [53,54,55,56,57,58,59,60]. Phase quantification analysis indicates that calcite constitutes approximately 93 ± 5 wt % of the crystalline material, confirming its role as the primary preparation layer component. The remaining 7 ± 2 wt % consists of minor phases including gypsum (CaSO_4_·2H_2_O) at approximately 6 ± 2 wt % and trace amounts of anhydrite (CaSO_4_) at roughly 1 ± 1 wt %. This compositional profile is entirely consistent with traditional Egyptian gesso preparation techniques [53]. The presence of minor gypsum (CaSO_4_·2H_2_O) and anhydrite (CaSO_4_) alongside dominant calcite requires interpretation. These calcium sulfate phases may reflect several possibilities: intentional addition of gypsum as a secondary component in the preparation mixture; natural occurrence as mineral impurities within the limestone; or post-depositional formation through reaction between calcite and sulfate-bearing burial environment components (groundwater, decomposition products, or atmospheric sulfur compounds) [76]. The exceptional purity and crystallinity of the calcite phase, indicated by the sharp, intense reflections in the XRD pattern, suggest high-quality source material and careful preparation processing. The substantial calcite content detected in all colored layers (20–45% across black, blue, green, yellow, and red decorations) confirms that pigments were applied over this calcium carbonate-rich ground, with sampling frequently capturing both the pigmented surface coating and portions of the underlying preparation layer [53,54,55,56,57,58,59,60].

The X-ray diffraction pattern of the gilded decoration (Figure 9b) reveals a ternary composition comprising metallic gold, calcite substrate, and an iron oxide component [61].

Phase quantification indicates that calcite remains the dominant crystalline phase at approximately 55 ± 5 wt %, reflecting the underlying preparation layer beneath the gold leaf. Metallic gold (Au) constitutes approximately 40 ± 2 wt % of the analyzed material, while hematite (Fe_2_O_3_) comprises roughly 5 ± 2 wt %. The gold reflections in the diffractogram appear at characteristic 2θ positions corresponding to the (111), (200), (220), and (311) crystallographic planes [77].

The detection of 5% hematite (Fe_2_O_3_) in the gilded area reflects the use of a colored preparation or mordant layer applied beneath the gold decoration. These colored underlayers served multiple technical and esthetic functions. The craftsmen would have applied a thin layer of red ochre mixed with binder over the white calcite preparation, allowed it to dry, burnished or smoothed the red surface, and then applied gold leaf or powder over this prepared substrate. The hematite detected in this XRD analysis confirms that this multi-layer gilding system was employed [78].

##### Advantages of SR-XRD

In general, SR-XRD demonstrated superior analytical performance compared to conventional laboratory XRD. Its low detection limit (<1 wt %) enabled the identification of minor deterioration phases, including residual gypsum (~6%) and trace anhydrite (~1%) in the investigated preparation layer. Such phases would remain undetectable using standard laboratory XRD, which typically has a detection limit of 3–5 wt % [79,80]. Moreover, SR-XRD requires only microgram-scale sample amounts (0.1–0.2 mg), in contrast to the milligram quantities (5–10 mg) needed for conventional powder diffraction [81]. This significantly minimizes damage to valuable artifacts while maintaining analytical reliability. In addition, the micrometer-scale spatial resolution of SR-XRD allows for the analysis of heterogeneous materials, such as paint layers, without mechanical separation, thereby preserving samples for further diagnostic investigations [81].

### 3.4. Conservation Approach

A hierarchical conservation approach is required to address the observed degradation patterns. Treatment prioritization should proceed as follows: (1) immediate mechanical stabilization of detached paint layers and desalination to arrest atacamite formation; (2) short-term wood consolidation with protective interfacial barriers; and (3) long-term environmental monitoring with periodic non-destructive reassessment to track degradation progression [82,83,84,85,86].

Consolidation treatments should include hydroxypropyl cellulose or low-molecular-weight polyethylene glycol (PEG 200–400) for cellulose stabilization, Paraloid B-72 (2–5% *w*/*v* in ethanol/toluene) for reversible paint layer consolidation, and methylcellulose (2–3% aqueous) or sturgeon glue for friable pigment cohesion [82,83,84].

Environmental control parameters must be maintained at a temperature of 18–20 °C (±2 °C), relative humidity of 45–55% (±5%), and light exposure <50 lux with UV filtration (<75 μW/lumen) [85,86].

## 4. Conclusions

This study demonstrates the effectiveness of integrating synchrotron XRD with XRF, FTIR, and confocal microscopy to analyze a Ptah–Sokar–Osiris wooden statuette from the 26th Dynasty, Giza. The multi-analytical approach enabled complete pigment identification, quantitative evaluation of wood deterioration, and characterization of post-burial transformations with minimal sampling.

Wood analysis confirmed the use of *Vachellia nilotica*, with synchrotron XRD showing a cellulose crystallinity index of 62.9%, indicating moderate structural preservation. FTIR revealed severe chemical damage, including a moderate carbonyl index (2.22) and loss of amorphous cellulose and hemicellulose, reflecting extensive lignin oxidation. Together, XRD and FTIR provided complementary insight into polymer degradation mechanisms in ancient wood.

Pigment analysis revealed an original, technologically sophisticated palette comprising carbon black applied over calcite-based preparation layers; Egyptian Blue (55 ± 5 wt %); malachite-based green pigments, now preserved only as minor remnants (10 ± 2 wt %); orpiment (60 ± 5 wt %); hematite (69 ± 5 wt %); and a high-purity calcite preparation layer (93 ± 2 wt %) with minor gypsum. The predominance of atacamite (65 ± 5 wt %) in the green layers is interpreted as a secondary, post-depositional transformation product formed through chloride-mediated alteration of the original malachite pigment during burial. The identification of metallic gold further confirms the elite quality of the object.

FTIR analysis revealed weak organic signals and an absence of protein amide bands. This pattern appears to be consistent with the use of a carbohydrate-based binding medium (such as a plant gum) rather than a proteinaceous one; however, it does not provide definitive identification. In a degraded, complex archeological matrix, the weak and non-specific nature of the remaining organic FTIR signals hinders a more conclusive characterization of the original binder, highlighting the limitation of this technique for such determinations without corroboration from more sensitive, molecule-specific yet destructive methods such as GC-MS.

The results highlight deliberate pigment mixing strategies and burial-induced alteration, especially the transformation of malachite into atacamite in chloride-rich conditions. The consistent presence of calcite across color layers indicates a standardized ground layer technology in Saite workshops.

Methodologically, the work demonstrates that synchrotron XRD can identify minor phases (<5%) from millimeter square samples, outperforming conventional approaches. Combined techniques enabled reliable distinction between original materials and post-depositional alteration.

Conservation implications are significant: severe binder loss, oxidative wood damage, chloride-driven mineral changes, and salt accumulation indicate the need for stabilization and controlled storage.

Beyond this object, the findings expand technical knowledge of Saite-period pigment traditions and provide reference baseline data for comparative studies. The workflow presented here offers a minimally invasive analytical framework for future research on polychrome wooden artifacts.

## Figures and Tables

**Table 1:** Description, location, and nature of analyzed samples.

Type of Samples	Description	No. of Samples
Painted layers	2 Black, 3 Blue, 1 Green, 1 Yellow, 2 Red, and White (Preparation Layer)	10
Wood	2 wood samples collected from the statuette and its base fallen parts	2
Gold leaf	1 gold sample was attached to the fallen preparation layer	1
Note	Duplicate samples of each color were collected from different locations on the object to capture variations in hue and intensity	

## Data Availability

The original contributions presented in this study are included in the article. Further inquiries can be directed to the corresponding author.
